# Spatial organisation of the mesoscale connectome: A feature influencing synchrony and metastability of network dynamics

**DOI:** 10.1371/journal.pcbi.1011349

**Published:** 2023-08-08

**Authors:** Michael Mackay, Siyu Huo, Marcus Kaiser

**Affiliations:** 1 Newcastle University, School of Computing, Newcastle upon Tyne, United Kingdom; 2 East China Normal University, School of Physics and Electronic Science, Shanghai, China; 3 University of Nottingham, NIHR Nottingham Biomedical Research Centre, School of Medicine, Nottingham, United Kingdom; 4 University of Nottingham, Sir Peter Mansfield Imaging Centre, School of Medicine, Nottingham, United Kingdom; 5 Shanghai Jiao Tong University, School of Medicine, Shanghai, China; École Normale Supérieure, College de France, CNRS, FRANCE

## Abstract

Significant research has investigated synchronisation in brain networks, but the bulk of this work has explored the contribution of brain networks at the macroscale. Here we explore the effects of changing network topology on functional dynamics in spatially constrained random networks representing mesoscale neocortex. We use the Kuramoto model to simulate network dynamics and explore synchronisation and critical dynamics of the system as a function of topology in randomly generated networks with a distance-related wiring probability and no preferential attachment term. We show networks which predominantly make short-distance connections smooth out the critical coupling point and show much greater metastability, resulting in a wider range of coupling strengths demonstrating critical dynamics and metastability. We show the emergence of cluster synchronisation in these geometrically-constrained networks with functional organisation occurring along structural connections that minimise the participation coefficient of the cluster. We show that these cohorts of internally synchronised nodes also behave *en masse* as weakly coupled nodes and show intra-cluster desynchronisation and resynchronisation events related to inter-cluster interaction. While cluster synchronisation appears crucial to healthy brain function, it may also be pathological if it leads to unbreakable local synchronisation which may happen at extreme topologies, with implications for epilepsy research, wider brain function and other domains such as social networks.

## Author summary

Significant research has investigated how the brain network, leads to diseases such as epilepsy. To date, most of this work has explored connectivity on the macroscale; between whole brain regions. Here we explore how the topology of mesoscale networks, between cortical columns, could affect functional behaviour. We use a model of a simple oscillator; the Kuramoto model, with a variety of networks to investigate how the network topology affects how these oscillators synchronise with each other. It is hypothesised that healthy brain operates at a critical point where regions can both synchronise and desynchronise with each other easily which is dependent on the strength of connections between nodes. We show that the topology dramatically affects this behaviour and that certain patterns allow this critical behaviour to exist over a wider range of connection strengths, making the system more robust. We show the oscillators synchronise in clusters, which themselves behave as if they were individual oscillators, and show interactions with other clusters. While this behaviour may be necessary for healthy brain function, at extreme topologies this may become pathological if it leads to unbreakable cluster synchronisation with implications for epilepsy research and other domains such as social networks.

## Introduction

Synchronisation is a widely studied phenomenon across a huge range of scientific domains and across an equally long period of time. One of the most widely used models in the study of synchronisation between coupled oscillators is the Kuramoto model [1,2]. The Kuramoto model describes a phase oscillator with an intrinsic frequency of oscillation which can interact with an arbitrary number of neighbours, and the change in its phase over time is dictated by its own intrinsic frequency and the relative phases of any interacting oscillators. Work involving Kuramoto oscillators has been broad and varied, and expanded over the years, with analytical solutions derived for systems of coupled oscillators [3].

As the field of network science has expanded in recent years, so too has work on the application of Kuramoto oscillators to networks [4–6]. The application of a simple model of synchronisation to networks allows an insight into how dynamical systems behave within a network and has seen application to as diverse fields as power grids [7,8], social networks [9], and neural synchronisation [10].

One of the properties which makes systems of coupled Kuramoto oscillators so widely studied is that they exhibit criticality [11]. A system of oscillators progresses through a phase transition as coupling strength increases, with the system as a whole moving from unsynchronised to synchronised behaviour. As the system transitions it passes through a critical point where oscillators are capable of both synchronising with coupled neighbours, but also desynchronising. Critical behaviour is posited as being vital in many systems, particularly biological systems [12,13], as it allows for dynamic correlation and facilitates information transfer [14]. This can be quantified with a property called metastability [15], which describes a system’s ability to transition between transient attractor-like states, and fluctuate between them.

Within the field of neuroscience there has been an explosion in the use of network science to probe the function of the brain in health and disease [16]. Communication within the brain is hypothesised to occur through synchronisation between neural populations [17], but complete synchronisation would fix the brain in one state, while no synchronisation would stop different parts of the brain communicating. For this reason, the brain is hypothesised to exist at a critical state, and there is a broad range of theoretical work and observational evidence to support this [14,18–22]. The Kuramoto model, as a model of synchronisation and criticality, is therefore well suited to exploring the dynamic behaviour of brain networks.

As the ability of the brain to transition fluidly between states is posited to underlie normal functioning of the brain, so too has disruption in this ability, which can be quantified by metastability, been hypothesised to result in brain dysfunction in disease [23–25]. This has led to application of the Kuramoto model to empirical networks of human brain in health and disease, or lesioned healthy networks, to probe whether a change in the functional behaviour of these models correlates with clinical features of disease [26–30].

Most of this work has been done on structural and functional networks derived from tractography and functional MRI (fMRI) respectively, which looks at brain networks on a region-to-region scale, or macroscale. However, particularly within the field of epilepsy research, there has been work looking at how network effects at the mesoscale level underpin epileptogenicity [31–33]. In addition, there is a growing body of empirical evidence that the spread of ictal activity occurs on a mesoscale, across the neocortex [34–37]. A limitation on studies exploring the mesoscale level of brain connectivity is simply the lack of empirical knowledge of the structure of neocortical connectivity at this scale. While microscopy can reveal the microcircuitry within a cortical column, and MRI can reveal large fibre bundles linking brain regions, there is no means of tracking the enormous number of connections between cortical columns made by fibres too small to be seen by MRI and too numerous to be identified by microscopy.

Our aim with this work was to therefore explore the effects of topology on the dynamical behaviour of a mesoscale model of neocortex. In doing so we will be investigating a spatially distributed network of nodes, where interaction between nodes is subject to a delay determined by the distance between nodes, and a simulated conduction velocity. Our interest was in how significantly the behaviour of the system was altered by changes in topology, and whether this gives any insights into how local topology could affect phenomena such as epileptogenicity or seizure spread.

## Methods

### Network generation

**Fig 1:**
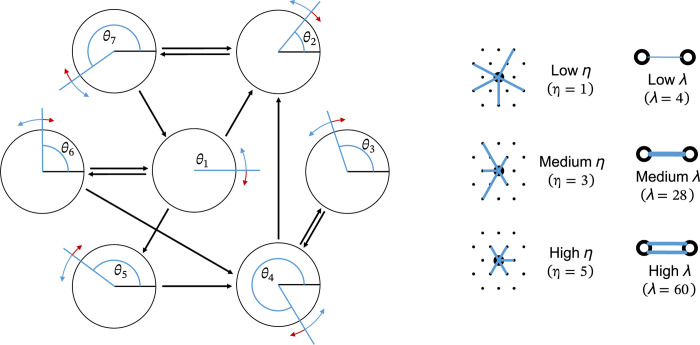
**a)** Kuramoto oscillators are spatially arranged in a hexagonal grid and are sparsely connected to each other. Each oscillator has a phase term, *θ*, which advances according to its own intrinsic frequency and its interaction with neighbouring nodes. **b)** Throughout the following figures we use the above icons to represent the topology and coupling strength of the simulated system.

Geometrically constrained, directed, unweighted, random networks were created by arranging 1600 nodes in a 40 by 40 hexagonal grid (Fig 1A), wrapped around a toroidal surface to avoid edge effects. The probability of an edge being generated between any two nodes *u* and *v* is given by:

$$P(u,v)={E(u,v)}^{-\eta }$$

Where *E* represents the Euclidian distance between two nodes. Edges are added to the network until reaching a total of 25600, giving an edge density of 1%. Edge density is held constant for every generated network. At low values of *η*, nodes make connections over a wide range of distances, while at high values nodes have a strong preference to make connections only with nearby neighbours.

### Kuramoto model

Each node is represented by a Kuramoto oscillator. The model is represented by a single variable; the phase of the oscillator, *θ*, and its evolution for a given oscillator *θ*_*i*_ is given by:

$${\dot{\theta }}_{i}=\omega_{i}+S_{i}(t)+\lambda \sum_{j=1}^{N}A_{ij}sin(\theta_{j}-\theta_{i}),i=1,\ldots ,N$$

Where *λ* represents the global coupling strength, *ω*_*i*_ represents the intrinsic frequency of the given oscillator, *A* represents the randomly generated adjacency matrix, and *S*_*i*_ represents stochastic noise. Each oscillator is randomly assigned an intrinsic frequency from a normal distribution with a mean frequency of 60Hz and standard deviation of 3Hz. Noise is included as a random phase deviation and per timestep is drawn randomly from a normal distribution with mean 0 and standard deviation of 0.04 radians.

### Simulations

Networks were randomly generated at values of *η* ranging from 1 to 5. Each node in the network is represented by a Kuramoto oscillator. The distance between nodes is set at 0.5mm, and the conduction velocity as 4m/s. These numbers were chosen as they represent an approximate distance between cortical columns in human neocortex [38], and a conduction velocity within the range of that of unmyelinated neurons [39,40]. To account for conduction delays the Kuramoto oscillators are simulated as:

$${\dot{\theta }}_{i}=\omega_{i}+S_{i}(t)+\lambda \sum_{j=1}^{N}A_{ij}sin(\theta_{j}(t-{dt}_{ij})-\theta_{i}(t)),i=1,\ldots ,N$$

Where for each node the phase *θ*_*i*_ is taken at the current timepoint, (*t*), while the phase of every other node, *θ*_*j*_, is taken at time (*t*−*dt*_*ij*_), where *dt*_*ij*_ is the conduction delay for the edge connecting nodes *i* and *j*. It is calculated using the conduction velocity above and the Euclidian distance between the nodes.

Simulations were run for 10 seconds, with a time step of 0.1ms using an Euler solver. For each value of *η* 16 different, random networks were created and for each network simulations were run over a range of 16 different coupling strengths. For each simulation the intrinsic frequencies of the nodes were randomised.

As shown in Fig 1B we use symbols throughout the rest of our figures to quickly represent whether simulations were run on networks with low, medium, or high values of *η* and *λ*. For *η*, 1 represents a low value, 3 a medium value and 5 a high value. For *λ*, we use 4 as a low value, 28 as a medium value and 60 as a high value.

To analyse the data, we calculate an order parameter, *r*, for the system as a whole, as the mean of the complex phase vectors of all the nodes at each time point:

$$re^{i\varphi }=\frac{1}{N}\sum_{j=1}^{N}e^{i\theta_{j}}$$

**Fig 2:**
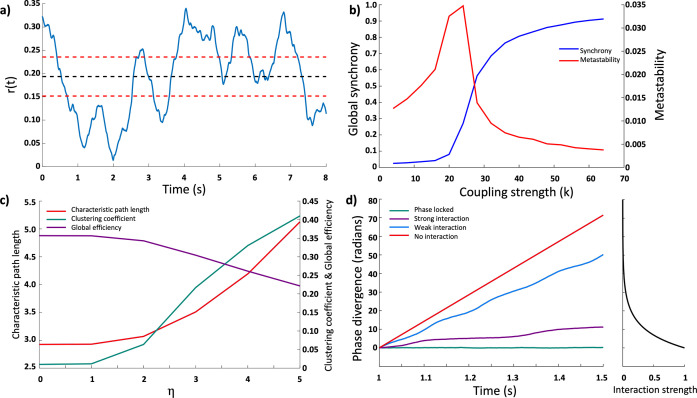
**a)** Progression of the order parameter of an example network over time, with the calculated synchrony and metastability shown as dotted black and red lines respectively. Metastability is shown as one standard deviation about the mean. **b)** The synchrony and metastability of a random network as a function of coupling strength. This shows the characteristic transition through a critical state, with a rapid increase in global synchrony accompanied by a peak in metastability. **c)** Graph theory measures of generated networks as a function of *η*. As *η* increases the characteristic path length and clustering coefficient increase, while the efficiency of the network decreases. **d)** Example of phase divergence over time between a pair of nodes for varying strengths of interaction (left). When the nodes are uncoupled their phases will diverge at a constant rate. As the interaction strength increases, this acts to slow down the rate of phase divergence, until the nodes are strongly coupled enough that they lock phases. We can therefore calculate the interaction strength based on the rate of phase divergence over a time window (right).

Where *φ* represents the mean phase of the system. The mean of the order parameter, *r*, over the whole simulation can be considered as the synchrony of the network, while the standard deviation of *r* represents the metastability of the network (Fig 2A). When simulations were run on a random network (*η* = 0) we see the characteristically described behaviour of a system of coupled Kuramoto oscillators, with a critical transition point as the coupling strength is increased, along with a peak in metastability (Fig 2B).

### Functional interactions

To examine the functional behaviour of the system we looked at pairwise interactions between nodes. When two Kuramoto oscillators have different intrinsic frequencies the difference between their phases increases over time. As the strength of interaction between these nodes increases, the rate of phase divergence decreases, until the point that they become phase locked (Fig 2D). We can use this phase divergence to estimate the strength of interaction between every pair of nodes using the equation:

$$I_{ij}(t)=e^{-|\delta \theta_{ij}(t)|}$$

Where *δθ*_*ij*_ is the change in phase difference between nodes *i* and *j* over a 100ms sliding window which is moved through the time series with a 50% overlap. At the critical coupling strength, the mean frequency difference between nodes was 1.3Hz. The window width was chosen to allow robust detection of phase differences between nodes. This creates an undirected, weighted network of functional interactions between nodes during each window which evolves with time.

To investigate whether there was any structure to the functional behaviour we used Newman’s spectral community detection algorithm [41,42] as implemented in the Brain Connectivity Toolbox [43], with a value of *gamma = 1*, to discern any modules in the functional networks for each time window. Modules were tracked over the series of time windows using an algorithm which looked for similarity between modules in subsequent windows. For each module in the previous time-step the module in the next time-step which shared the greatest overlap of member nodes was assigned the same identifier. This allowed us to track modules over time as member nodes joined and left.

## Results

### Synchronisation and metastability in sparse networks

**Fig 3:**
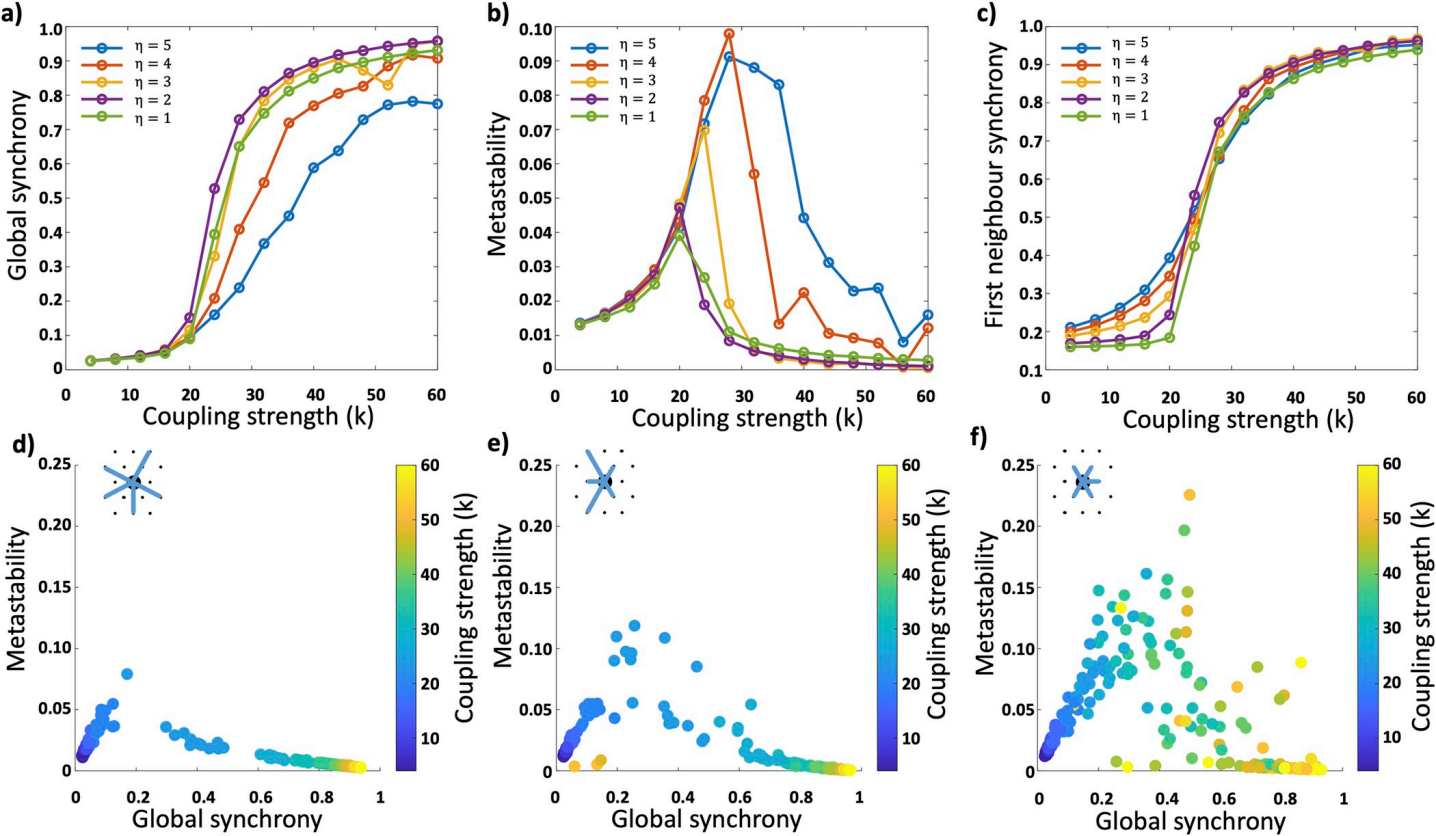
Synchrony and metastability with varying network topology. **a)** As *η* is increased, networks resist global synchrony and show a disruption in the transition through criticality. **b)** This disruption manifests as a dramatic increase in overall metastability which occurs over a much wider range of coupling strengths. **c)** In networks with a low value of *η*, nodes only synchronise with their immediate neighbours at the critical coupling point and beyond. As *η* is increased nodes start to synchronise with their immediate neighbours well before the critical point and the emergence of any global synchronisation. **d-f)** Plots of global synchrony against metastability at different coupling strengths for three different topologies; *η = 1 (d)*, *η = 3 (e)*, and *η = 5 (f)*. At higher values of *η*, for an increasing range of coupling strengths, networks show dramatically higher metastability and a fundamentally different relationship between global synchrony and metastability.

We initially looked at the simulation results in the context of previously described behaviour of Kuramoto oscillators in complex networks (Fig 3A and 3B). At low values of *η* we see the classical behaviour of Kuramoto oscillators; an initially low global synchrony, which, as coupling strength increases, passes through a critical transition point where the synchrony increases rapidly, and we see a peak in metastability. The synchrony then plateaus off, and metastability falls to very low values as the coupling strength increases further.

For networks with a high value of *η*(4≤*η*≤5) as coupling strength increases there is a resistance to global synchrony. There is no sharp transition point and the metastability of the network continues to rise to significantly higher levels over a wide range of coupling strengths. This additionally manifests itself when looking at the average synchrony of nodes with their immediate neighbours (Fig 3C). In low *η* networks, there is very little change in local synchrony until reaching the critical coupling strength, followed by an explosive onset of synchrony. As *η* increases there starts to be a noticeable increase in local synchrony at coupling strengths significantly lower than the critical transition point; meaning nodes are synchronising with their immediate neighbours before the onset of any global synchrony. This is in line with previously described results [6].

What was also very apparent was the extreme heterogeneity of the behaviour of the systems in the high *η* networks. In low *η* networks, simulations run on different networks produced very similar values of global synchrony and metastability across all coupling strengths (Fig 3D). In high *η* networks, at coupling strengths beyond the critical transition strength, the calculated values of global synchrony and metastability were highly variable from simulation to simulation, and across a wide range of coupling strengths. This variability increased with increasing *η* (Fig 3E and 3F).

### Emergence of functional organisation

In order to investigate this extremely metastable and heterogeneous behaviour we looked at the functional behaviour of the system, deriving a temporally-evolving functional connectivity matrix based on pairwise synchrony between nodes over a sliding time window. We used Newman’s spectral community detection algorithm to detect the presence of any sets of nodes which were acting as a functional community and tracked these modules over the series of time windows.

**Fig 4:**
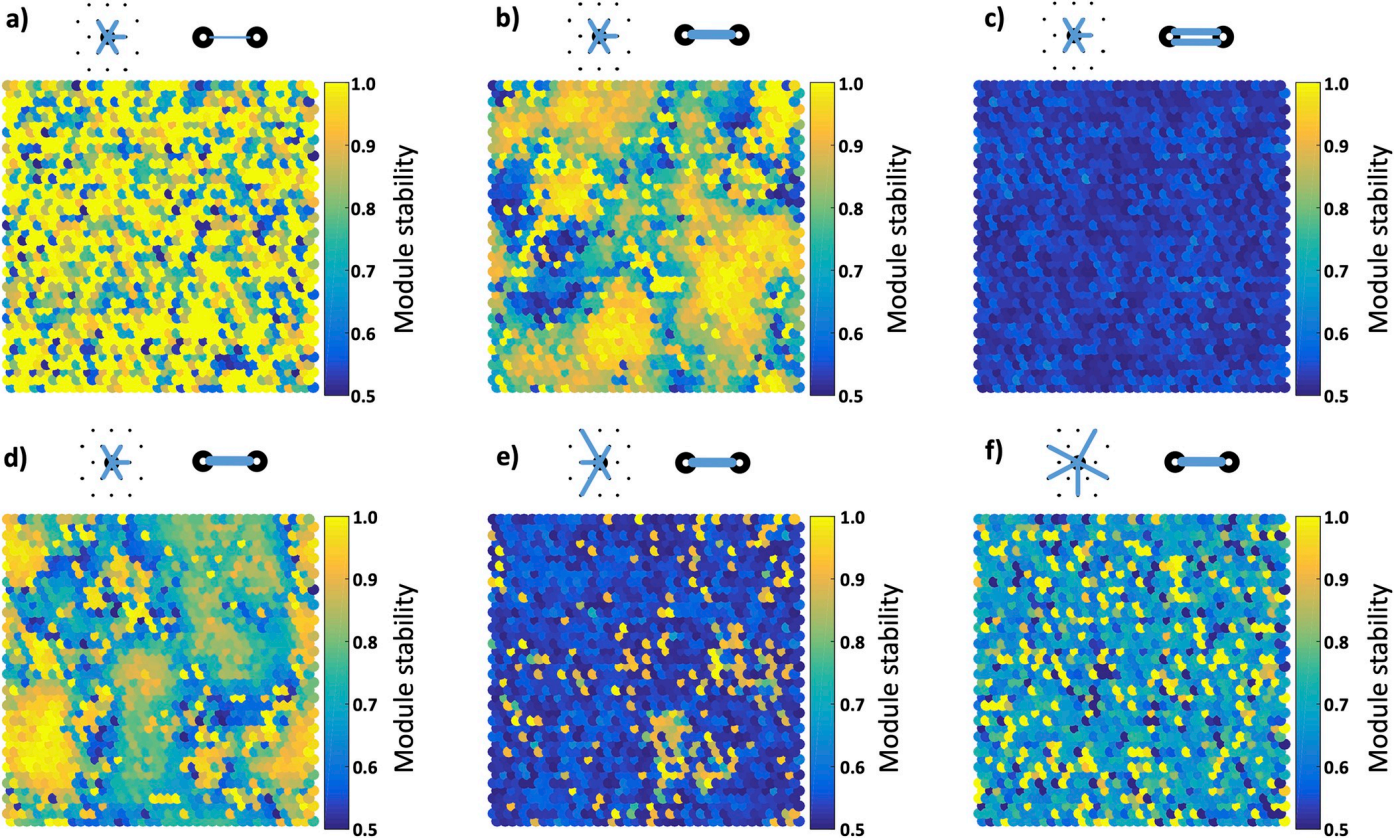
Module stability and organisation is related to coupling strength and topology. **a-c)** Module stability (the proportion of time a node spends in its most frequently associated module) in a high *η* network as coupling strength is increased. At low strengths the lack of interaction between nodes mean they tend to remain in the same functional module over the whole simulation (a). At high strengths the network is largely synchronised with nodes randomly being assigned into modules, and this results in a nodes assignment frequently changing giving a low stability (c). At coupling strengths close to but greater than the critical coupling strength we see spatially organised groups of nodes which belong to the same module and tend to remain in the same module over time giving clusters of high stability (b). These areas are separated by nodes which are metastable, transitioning between one module and the other over time. **d-f)** Module stability at the critical coupling strength as *η* is varied. As *η* is reduced, the spatially organised modularity seen in the high *η* networks (d) disappears (e-f).

At low coupling strengths, nodes are stable within their module as there is very little interaction between nodes (Fig 4A). At high coupling strengths the global synchrony leads to detection either of the whole sheets as a single module, or modules being detected as a random subset of the whole network, with a low modularity (Fig 4C). In high *η* networks, for moderate coupling strengths greater than the critical coupling strength, modules emerge with a relatively stable core of nodes which are organised spatially. These stable areas are surrounded and separated by areas which are less stable; that is the nodes are switching between modules more frequently (Fig 4B). This behaviour does not occur in the low *η* networks, with a rapid transition from the uncoupled systems to globally synchronised systems (Fig 4E and 4F).

In supplementary S4 Fig we show the aggregate behaviour of nodes in more detail. As a node is assigned to a module within each time window, we could look at the number of assignment changes happening on average from window to window. In all networks there is a pattern of a low rate of assignment changes at low coupling strengths gradually increasing to a high rate at high coupling strengths. The high η networks however show a significant dip in the average rate of assignment changes as they transition through the critical coupling strength reflecting the organisation of stable modules.

To look at the organisation of these modules we calculated a variant of the participation coefficient for each node using the structural connections and the modules derived from the functional network:

$${PC}_{i}=\frac{k_{i}^{external}}{k_{i}^{total}}$$

**Fig 5:**
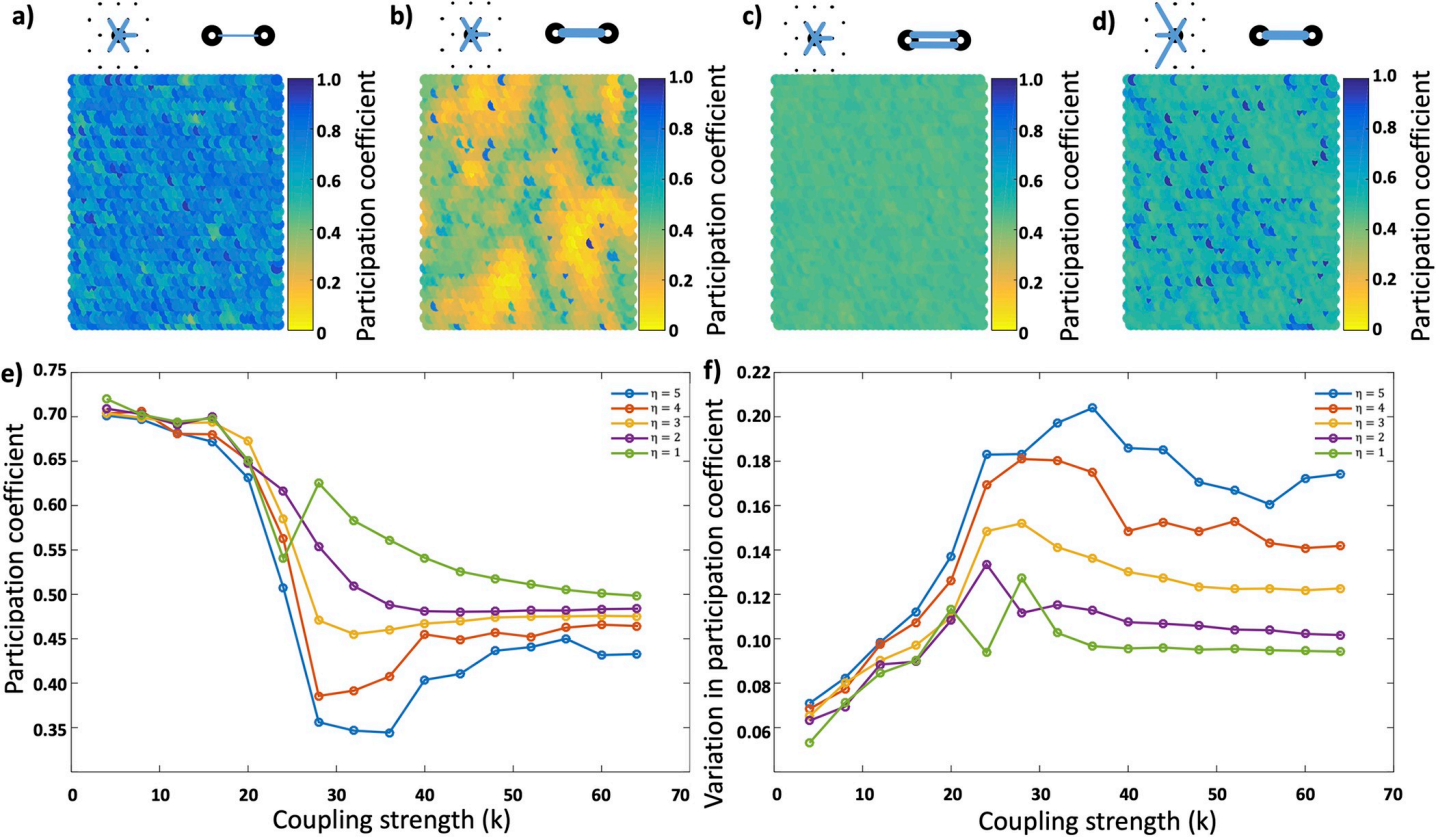
**Functional behaviour of networks; a-d)** Participation coefficient in high *η* networks at a range of coupling strengths (*a-c*) and in a low *η* network at the critical coupling strength (d). At critical coupling strengths in high *η* networks (*b*) functional modules emerge which synchronise internally, resulting in a low participation coefficient, and which organise spatially. **e)** Average participation coefficient of all nodes in the network as a function of coupling strength and topology. Participation coefficients fall during the transition through the critical coupling strength but fall significantly more in high *η* networks. **f)** The average variation in participation coefficients of individual nodes over time. High variance suggests nodes are dropping in and out of functional modules to a greater extent and therefore exhibiting greater metastability.

Nodes with a low participation coefficient therefore make proportionally more connections with nodes which are part of the same functional module. Participation coefficients are typically high at both low and high coupling strengths (Fig 5A–5C), however in high *η* networks subsets of nodes reach particularly low participation coefficients. These nodes are again spatially organised and correspond to the previously described nodes which have a stable functional module (Fig 5B).

In addition to average participation coefficients being minimised in high *η* networks and at coupling strengths beyond the critical coupling point, we also see an effect on the variability of the participation coefficient (Fig 5E and 5F). In high *η* networks a node’s participation coefficient is more variable over the time series, reflecting its increased propensity to drop in and out of its functional module, and therefore demonstrating the metastability of the network. It is relevant to note that the spatial organisation is a result of the geometric nature of the random network, and the organisation, as shown by the participation coefficient, is a product of the topology of the network. When we randomly shuffle the nodes and repeat the simulation with the shuffled adjacency matrix we lose the spatial organisation but continue to find nodes with low participation coefficients.

We confirmed the special organisation quantitatively and show this in S4F Fig, where we take the centre of mass of each module and then calculate the average distance of each node from the centre of mass of its assigned module. While this distance remains stable and high for low η networks, as η is increased we see larger and larger falls in the average distance through the critical coupling strength reflecting the spatial clustering of modules.

### Modules mimic the behaviour of weakly coupled nodes

**Fig 6:**
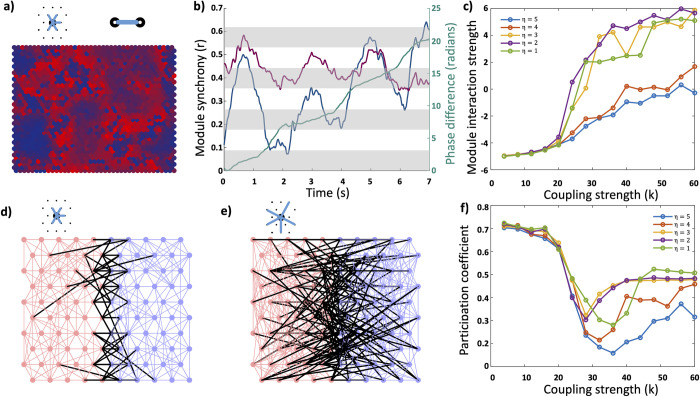
**Inter-module interaction; a)** Example network with two functional modules with the colour representing the proportion of time each node spends within a module. **b)** The internal synchrony of the two modules (*red and blue)* and the difference in the phase of the mean field of each module (*green*). One module has a mean field frequency of 60.13Hz, and the second a frequency of 60.51Hz. The phase difference progresses in a similar manner to two weakly coupled single oscillators, with slow divergence at a phase difference of 0 to *π* radians, and a rapid divergence from *π* to 2*π* radians. We have shaded the background for each block of *π* radians to highlight this pattern of phase divergence. The rapid divergence is coincident with dramatic internal desynchronisation and resynchronisation events within the modules. **c)** The rate of phase divergence can be used to calculate the strength of interactions between modules. In low *η* networks, there is a rapid transition to strongly coupled (and therefore synchronised) modules with increasing coupling strength. In high *η* networks the modules remain weakly coupled over a wide range of coupling strengths. **d-e)** Illustrative networks demonstrating that for low *η* networks (*e*) we would expect to see a higher participation coefficient for modules and therefore greater interaction. **f)** Plot of the average participation coefficient of modules as a function of coupling strength and topology demonstrating significantly lower participation coefficients of modules forming in the high *η* networks over a wide range of coupling strengths beyond the critical coupling strength.

We next looked at the behaviour of these functional modules. By assigning each node into the module it was most frequently part of over the course of the simulation, we could calculate the order parameter and mean phase for each module independently. We found that when this organised behaviour emerges in high *η* networks, the modules behave as if they were an individual Kuramoto oscillator; with their own intrinsic frequency, and interactions between modules (Fig 6B). This leads to a state where parts of the system are highly synchronised, but asynchronous with the rest of the system, as the modules behave independently. The interaction between modules causes a familiar pattern of slow drift of the relative mean phases of the modules while they are between 0 and *π* radians apart, followed by a rapid phase drift between *π* and 2*π* radians. The period of attraction between the modules is associated with high internal synchrony within modules. As the modules reach the point at which their mean phases cause repulsion there is rapid internal desynchronisation within the modules as the mean phase separation quickly increases, before resynchronising internally.

We see this reflected in node assignment behaviour in S4B Fig. As the modules desynchronise there is a peak in the rate of module assignment changes, and then during the attraction period the modules stabilise, and the rate of assignment changes drop. This is reflected in S4E Fig showing a peak in the variance of the rate of assignment change in high η networks at the critical coupling strength.

As with the pairwise interactions between nodes, we can calculate the strength of the interaction between modules as a function of the rate at which the mean phase of the modules diverges (Fig 6C). In low *η* networks as the coupling strength passed the critical coupling threshold there is a rapid increase in the strength of interactions between modules, while in the high *η* networks there is a much more gradual increase. This means that the modules remain weakly coupled over a wide range of coupling strengths and likely is what allows the asynchronous behaviour of functional modules. When the modules are strongly coupled, like two strongly coupled nodes, they will synchronise.

This result fits with what would be expected based on the topology (Fig 6D and 6E). In high *η* networks connections are only made over short distances; as such connections between nodes in different modules only occur on the border between those modules. This means that only a small proportion of the connections to or from nodes in a module link to nodes external to that module. By contrast in the low *η* networks because nodes make longer distance connections significantly more nodes within a module will make connections with nodes external to the module. We show example networks from our simulations in the supplementary materials in S3 Fig with nodes coloured by module and intra-module connections coloured the same colour, while inter-module connections are coloured black. We can visualise the spatial organisation in the high η networks at critical coupling strengths and it is apparent that there are noticeably fewer inter-module connections as a proportion of the total connections. We can calculate a participation coefficient using the functional modules derived from our simulations and the underlying structural networks:

$$PC=\frac{external}{external+internal}$$

We calculate the proportion of structural connections from one functional module to another relative to the total number of connections made by nodes within that functional module, for every network simulated. We find that as the coupling strength reaches the critical coupling point there is a sudden decrease in *PC* as synchronisation starts to occur between connected nodes and modules start to organise. This decrease in *PC* is significantly larger and occurs over a wider range of coupling strengths in high *η* networks as expected, fitting with the hypothesis that it is the low participation coefficient which allows the modules to remain weakly coupled and resist global synchronisation.

The difference in mean field phase between all pairs of modules over the latter half of simulations (to remove any effect of initial transients) highlights the effect of delays in the slightly off 0 phase difference between modules seen at the critical coupling strength in low *η* networks (S5 Fig). Moreover, for high *η* networks at the critical coupling strength we do see peaks of activity centred around π/2 and 3π/2 representing peaks of the sine function and thus the phase difference at which there is the greatest interaction strength between modules.

## Discussion

What we see in this work is an example of cluster synchronisation, whereby discrete groups of nodes within a network synchronise internally with each other, and distinctly from the rest of the network. This is an area of research which has received significant attention recently due to its intuitive relationship with neural function [44]. The brain is hypothesised to exist in a critical state, allowing rapid synchronisation and desynchronisation of clusters of neurons, facilitating information transfer, and this necessitates local clusters of neurons to be able to synchronise independently of the rest of the network.

Recent work has focussed on describing a theoretical basis for the emergence and stability of such clusters [45–49], suggesting they emerge as a result of strong intra-cluster and weak inter-cluster connections as well as similarity in frequencies of oscillators within each cluster [50]. Much of this work has been theoretical, in small systems, and systems with inherent structure, and we replicate much of the findings here.

There are however notable implications from this work. Firstly, we have looked at networks which are spatially arranged, with delays in transmission between nodes, in line with real-world networks. The networks we have looked at have been entirely random, with no structure or modularity imposed on them and are neither scale-free nor small world. What stood out in early exploration of the network morphospace [51], where we grew networks which included preferential attachment terms, was that the induction of scale-free or small world properties in the networks made little functional difference in comparison to the distance attachment term. Hence our focus within this paper on manipulating this property alone. This was also reflected in early simulations where we looked at varying conduction delays, including systems with no delays. Increasing conduction delay causes a network to behave as if it had a slightly higher value of η but did not qualitatively change the behaviour seen in our results. We have shown the results from simulations without time delays in the supplementary materials in S1 and S2 Figs.

Given the importance placed on metastability in efficient cognitive function it is notable that the effect of increasing *η*, and thus increasing the clustering coefficient and path length and decreasing efficiency, had the effect of both significantly increasing metastability and widening the range of coupling strengths over which it was raised. This would suggest these networks are much more robust to changes in coupling strength while maintaining the ability to function at a critical point, with an ability to locally synchronise and form coalitions.

As we move to explore the functional behaviour with changing topology, we can see the emergence of functional clusters; sets of nodes which synchronise closely with each other but not with the rest of the network. This behaviour only emerges around the critical coupling strength of the system, and only in networks with *η*≥4. The clusters are spatially arranged; that is nodes which are close together are more likely to belong to the same module. A proportion of the nodes within a cluster remain stably within the cluster, while nodes which border clusters are more likely to switch between clusters repeatedly during a simulation. This results in the high degree of metastability seen in these simulations, as a significant proportion of the nodes in the network as a whole are recurrently switching synchronisation between different clusters.

In Figs 4 and 5 we can see the relationship between module stability and participation coefficient. We calculate the participation coefficient for each node as the proportion of structural connections from that node which connect to nodes in different functional clusters. A direct comparison between Figs 4B and 5B showing the same simulation demonstrates that nodes which remain stable within one cluster also show the lowest participation coefficient–that is they connect predominantly with nodes within the same cluster. As we move onto Fig 6 we calculate the participation coefficient for each cluster as a whole; of all the nodes within a cluster what proportion of their total connections are made to other clusters. Here we highlight a fascinating feature of these networks where we see the participation coefficient of the networks fall as coupling strength increases and we transition through the critical coupling strength. This represents the functional behaviour of the networks organising along structural connections, and stable clusters forming among nodes which minimise the participation coefficient.

By looking independently at the clusters within the networks over time we can also show the dynamic and metastable behaviour of these clusters. By calculating the mean phase of each cluster, we can analyse how the clusters interact with each other and show that the cluster as a whole behaves much like an individual node and they show weak coupling to neighbouring clusters. Each cluster has its own mean phase and intrinsic frequency, which proceeds relative to other clusters and shows gradual phase divergence from other clusters.

Two weakly coupled Kuramoto oscillators show a characteristic pattern of phase divergence. When their phases are between 0 and π radians apart there is a slow phase divergence, as the sine of the phase differences acts to slow the phase progression of the faster node and speed up the progression of the slower node–it acts to make the nodes converge in phase. As the phase difference exceeds π radians, the sine of the difference now acts to speed up the faster node, and vice versa, acting to make the phases diverge and leading to rapid phase change until the phase difference returns to 0 radians. We can see in Fig 6B the same pattern of phase divergence between two clusters, demonstrating the clusters as a whole behaving as weakly interacting nodes.

In addition, at the point that the behaviour transitions to repulsion and we see the rapid phase divergence between clusters there is a simultaneous rapid internal desynchronisation within the clusters. Given the stability of some nodes within clusters this can be explained as the bulk of the metastable nodes desynchronising from the cluster. As the phase difference between the clusters returns to the range 0 - π radians, this is associated with internal resynchronisation and the metastable nodes joining the same or a different cluster to previous.

Again, fitting with the idea that these more geometrically constrained networks show more robust critical behaviour we can calculate the interaction strength between clusters and show the weak coupling emerging at the critical coupling strength but then only very gradually increasing over a wide range of coupling strengths. In contrast the low *η* networks show rapid increase in cluster interaction strength, and hence global synchronisation.

Considering these results from the perspective of a model of neocortex it highlights the importance of network topology in allowing healthy neural functioning. Geometrically constrained connections are both biologically more cost-efficient, but also allow local synchronisation, and critical dynamics over a wider range of coupling strength resulting in a more functionally robust network. We know most real-world networks, and particularly brain networks show small-world and scale-free properties [52,53]. We would like to extend this world by exploring cluster synchronisation in these networks with a preferential attachment term to confirm the behaviour seen here. Intuitively we would expect little difference as only a very small proportion of these networks need to be rewired to induce small-world properties, but this will not significantly change the participation coefficients of large coalitions of nodes. Indeed, provisional results performed in early simulations support this.

A further avenue of research would be to explore the concept of external control of synchronisation. If such a sheet of neocortex operates at a critical point such that nodes can easily synchronise and desynchronise to transfer information, then it suggests that such a network should be susceptible to synchronising with an external input applied to a set of nodes. If we consider rapid global synchronisation of the whole network to be pathological–we need metastability in healthy brain function–then high *η* networks may also become pathological if they entrain stable, local synchronisation that is robust against external input. In contrast to a generalised network problem, this would be a focal network disorder. What would make this more difficult to recognise in existing work is that the networks would likely still demonstrate high metastability due to interactions between clusters and the behaviour of large cohorts of nodes at the boundaries between clusters. Exploring the ability of external input to break cluster synchronisation would be important to help elucidate at what point increasing *η* results in pathological functional behaviour, where a network will generate an area of local synchrony which is persistent and unbreakable.

While this work is theoretical it also raises hypotheses subject to investigation in real world data. In the brain, particularly in a disease such as epilepsy, it presents questions as to whether subtle changes in connectivity at the mesoscale underlie epileptogenicity. In acquired epilepsy following TBI or stroke, is abnormal rewiring leading to topological changes which result in abnormal synchronisation, either by increasing the distribution of edge length and therefore predisposing to strong synchrony over a wide area or is rewiring extremely local resulting in a very high clustering coefficient and unbreakable local synchronisation. Could similar processes underlie the epileptogenicity of cortical dysplasia or dysembryoplastic neuroepithelial tumours?

Outside the brain there are implications in social networks. Much is made of the existence of echo chambers in social networks, which much discourse aimed at the use of algorithms and confirmation bias as contributing to their formation [54,55]. Yet consensus of opinion within groups of users has been modelled as a synchronisation phenomenon [56,57]. Equally, estimates of the local clustering coefficient within social networks such as Flickr and Live Journal, are placed at around 0.35 [58], right in the range of our high *η* networks. It may simply be a phenomenon that within highly clustered networks local synchronisation will happen spontaneously if there is sufficient strength of interaction between participants. It may also be that sufficiently extreme local topology may render such areas prone to local synchronisation that cannot be broken by external influence. Ultimately, further research on cluster synchronisation and robustness of local synchronisation may prove a fruitful avenue of research in brain and social networks.

## Supporting information

S1 Fig Synchrony and metastability as a function of network topology in simulations with no time delays. A reproduction of Fig 3 based on simulations run with no time delay. We see qualitatively almost identical behaviour to the simulations with delays. High η networks show resistance to global synchrony with much higher metastability maintained over a wide range of coupling strengths and significant local synchrony below the critical coupling strength.
(PNG)

S2 Fig Participation coefficient as a function of coupling strength and η in simulations without time delays. **a)** As seen in Fig 5E, the behaviour where we see high η networks transitioning through the critical coupling strength and arranging along structural connections leading to low participation coefficients is seen identically in simulations without time delays. **b)** The low participation coefficient is correlated with a decrease in the mean distance of member nodes of a module to its centre of mass, showing the module becoming confined in space.
(PNG)

S3 Fig Community structure in high and medium η networks as a function of connection strength. In single simulations a timepoint is taken and the network is shown with each node coloured according to the module it belongs to at that time point. Structural connections between nodes belonging to the same module are coloured the same as the nodes. Connections between nodes in different modules are shown in black. The connection strength varies from low to high, from left to right. **a-c)** Show high η networks, **d-f)** show medium η networks. In **b)** which shows a high η network at the critical coupling strength we see the modules have clustered in space (particularly relative to **a** and **e**), and there is only a relatively small number of black inter-module connections reflecting a low participation coefficient. Lower η networks show a much higher proportion of black inter-module connections, as do the high η networks at extreme connection strengths.
(PNG)

S4 Fig Module assignment changes and spatial organisation. **a-c)** Here we show an η = 5 network at three different coupling strengths, 4, 28 and 60. In green we show the phase divergence between the two largest modules, in blue we show the total number of nodes which change module assignment within that time window. Of note, in **b** we see the pattern of weakly interacting modules with slow then fast phase divergence. There are peaks of nodes assignment changes which occur shortly after a fast divergence phase at a time when the modules are internally resynchronising. **d-e)** show the mean and standard deviation of the number of assignment changes over a whole simulation as a function of connection strength for a range of η. **f)** Shows the mean distance of modes from the centre of mass of their assigned module as a function of connection strength and η. In high η networks, through the critical coupling point, the mean distance decreases showing that modules are clustering spatially.
(PNG)

S5 Fig Distributions of inter-module phase differences as a function of topology and coupling strength. By rows, high η networks at the top to low η networks on the bottom row. By columns, low coupling strengths, then critical and high coupling strengths from left to right. Distribution of absolute phase differences between detected modules over latter half of simulations to exclude initial transients. At low coupling strengths, there is a uniform distribution of phase differences reflecting the lack of interaction between modules. At the critical coupling strength, in the high η network, you can see that modules spend most time with a phase difference around π/2 and 3π/2, which reflect the peaks of the sine function–and thus the period of greatest interaction strength. As η reduces there is a push towards synchrony and the modules spend most of the time in a very narrow phase range with peaks centred just off 0 and 2π radians, reflecting the effect of delays. At the maximum coupling strength these peaks of phase difference move to 0 in the low η networks, while the high η networks mimic the low η networks at lower coupling strengths.
(PNG)

S1 Code Source code used to produce the results and analyses. (ZIP)
