# Supplementary figures and images for: Spatial organisation of the mesoscale connectome: A feature influencing synchrony and metastability of network dynamics
Source: PLoS Comput Biol. 2023 Aug 8;19(8):e1011349. doi: 10.1371/journal.pcbi.1011349 (PMC10437862; doi:10.1371/journal.pcbi.1011349)

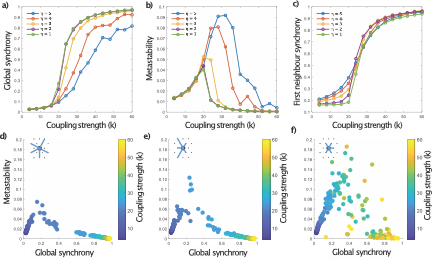

Supplement: S1 Fig — A reproduction of Fig 3 based on simulations run with no time delay. We see qualitatively almost identical behaviour to the simulations with delays. High η networks show resistance to global synchrony with much higher metastability maintained over a wide range of coupling strengths and significant local synchrony below the critical coupling strength. (PNG) [file pcbi.1011349.s001.png]

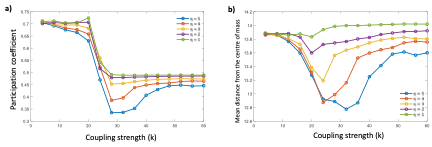

Supplement: S2 Fig — a) As seen in Fig 5E, the behaviour where we see high η networks transitioning through the critical coupling strength and arranging along structural connections leading to low participation coefficients is seen identically in simulations without time delays. b) The low participation coefficient is correlated with a decrease in the mean distance of member nodes of a module to its centre of mass, showing the module becoming confined in space. (PNG) [file pcbi.1011349.s002.png]

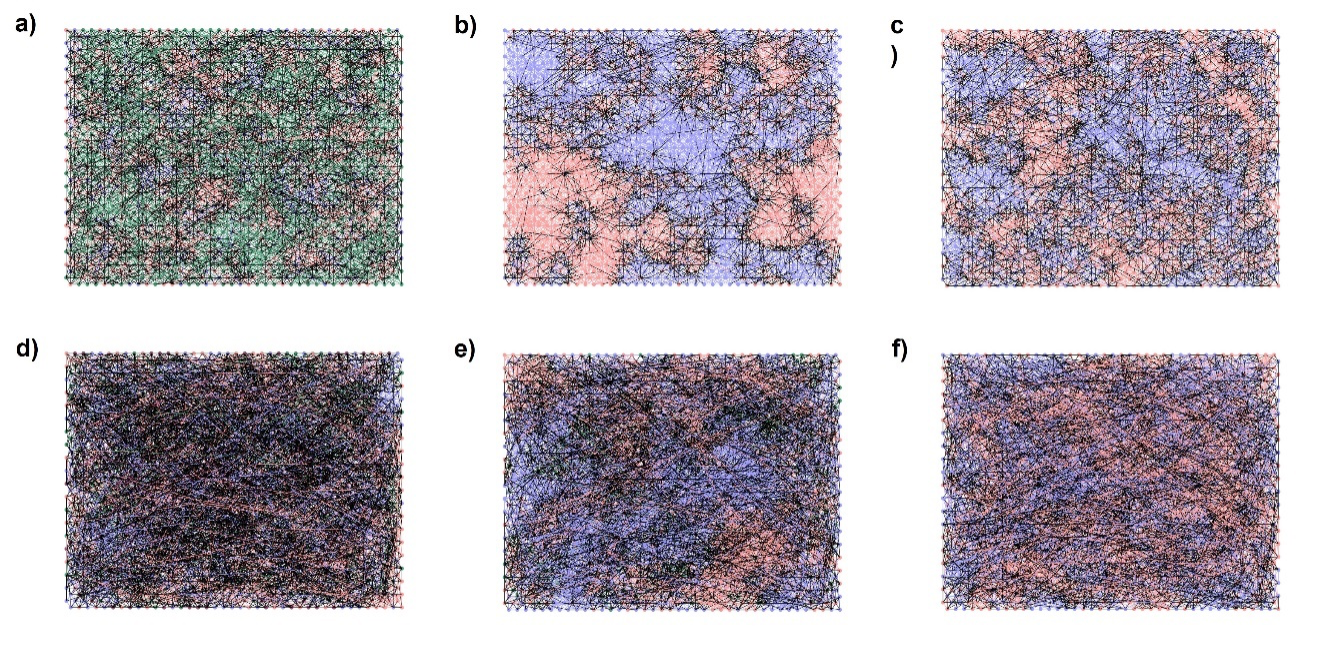

Supplement: S3 Fig — In single simulations a timepoint is taken and the network is shown with each node coloured according to the module it belongs to at that time point. Structural connections between nodes belonging to the same module are coloured the same as the nodes. Connections between nodes in different modules are shown in black. The connection strength varies from low to high, from left to right. a-c) Show high η networks, d-f) show medium η networks. In b) which shows a high η network at the critical coupling strength we see the modules have clustered in space (particularly relative to a and e), and there is only a relatively small number of black inter-module connections reflecting a low participation coefficient. Lower η networks show a much higher proportion of black inter-module connections, as do the high η networks at extreme connection strengths. (PNG) [file pcbi.1011349.s003.png]

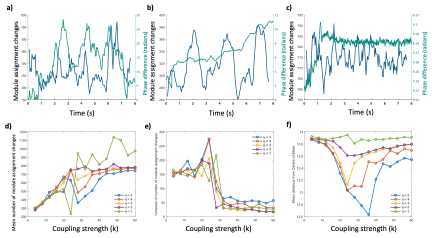

Supplement: S4 Fig — a-c) Here we show an η = 5 network at three different coupling strengths, 4, 28 and 60. In green we show the phase divergence between the two largest modules, in blue we show the total number of nodes which change module assignment within that time window. Of note, in b we see the pattern of weakly interacting modules with slow then fast phase divergence. There are peaks of nodes assignment changes which occur shortly after a fast divergence phase at a time when the modules are internally resynchronising. d-e) show the mean and standard deviation of the number of assignment changes over a whole simulation as a function of connection strength for a range of η. f) Shows the mean distance of modes from the centre of mass of their assigned module as a function of connection strength and η. In high η networks, through the critical coupling point, the mean distance decreases showing that modules are clustering spatially. (PNG) [file pcbi.1011349.s004.png]

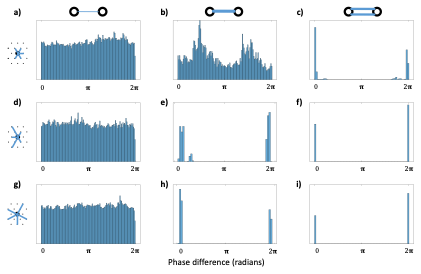

Supplement: S5 Fig — By rows, high η networks at the top to low η networks on the bottom row. By columns, low coupling strengths, then critical and high coupling strengths from left to right. Distribution of absolute phase differences between detected modules over latter half of simulations to exclude initial transients. At low coupling strengths, there is a uniform distribution of phase differences reflecting the lack of interaction between modules. At the critical coupling strength, in the high η network, you can see that modules spend most time with a phase difference around π/2 and 3π/2, which reflect the peaks of the sine function–and thus the period of greatest interaction strength. As η reduces there is a push towards synchrony and the modules spend most of the time in a very narrow phase range with peaks centred just off 0 and 2π radians, reflecting the effect of delays. At the maximum coupling strength these peaks of phase difference move to 0 in the low η networks, while the high η networks mimic the low η networks at lower coupling strengths. (PNG) [file pcbi.1011349.s005.png]
